# Acculturation strategies and blood cortisol in colombian Migrants in Chile

**DOI:** 10.1186/s40359-023-01147-w

**Published:** 2023-03-31

**Authors:** Alfonso Urzúa, Diego Aragón, Rodrigo Landabur, Diego Henríquez, Leonel Cortés

**Affiliations:** 1 Escuela de Psicología, Universidad Católica del Norte, Antofagasta, Chile; 2 Escuela de Medicina, Universidad de Tarapacá, Arica, Chile; 3 Departamento de Psicología, Universidad de Atacama, Copiapó, Chile; 4 Departamento de Tecnología Médica, Universidad de Antofagasta, Antofagasta, Chile

**Keywords:** Acculturation, Cortisol, Migration

## Abstract

**Background:**

migration is a worldwide phenomenon that is growing at an accelerated pace. When people who migrate come into contact with a new culture, they are immersed in a process called acculturation. In this process, people oscillate between maintaining their own culture or acquiring the culture and customs of the host country, resulting in the so-called acculturation strategies. According to Berry’s proposal, there are four main acculturation strategies: assimilation, integration, marginalization and separation. The few existing studies of Latinos in an Anglo-Saxon country relate the use of the integration strategy (biculturalism) with lower cortisol levels. No studies have been found on the subject in Latino migrants in a Latino country.

**Method:**

a cross-sectional design was used to analyze the relationship between acculturation strategies and blood cortisol levels, based on the hypothesis that an integration strategy or biculturalism would be linked to lower cortisol levels. The study involved 314 Colombian migrants in Chile, who were evaluated with a scale of acculturation strategies according to the model proposed by Berry, in addition to providing blood samples to analyze cortisol levels.

**Results:**

migrants who show a preference for leave behind the culture of the country of origin have higher levels of cortisol ng/ml in blood. According to multiple comparisons the mean cortisol value was significantly different between integrated and assimilated subjects, with the mean cortisol of the integrated being significantly lower than that of the subjects typed as marginalized.

**Conclusion:**

The patterns of the relationship between biculturalism and cortisol found in Latino migrants in Anglo-Saxon countries are repeated in Latino migrants in a Latino country. It is necessary to explore the influence of other variables in this relationship, since it seems that the best adaptive strategy, and therefore the cortisol response, will vary according to the socio-cultural context of the host country.

## Background

Migration is a worldwide social phenomenon, with an estimated 281 million international migrants by 2020, or around 3.6% of the world’s population. South American countries, including Chile, have not been immune to these processes, with an estimated 14.2 million migrants living in Latin America and the Caribbean as of 2020 [1].

There is abundant evidence that the migration process can have an impact on both physical and mental health, as well as on the well-being of the migrant, which can be mediated or moderated by variables of different types, which can mitigate or increase the negative effect that certain phenomena such as discrimination or stress can have on health and well-being. Some of the variables that have been found to have a protective effect on health in the migrant population living in the country are self-esteem, identity, absence of negative affect, social interaction, rootedness and acculturation strategies, among others.

Acculturation strategies correspond to interaction patterns, where the behavior of a migrant in contact with the host environment may be influenced by the maintenance of the customs, uses and identity of the migrant’s own culture versus the adoption of those of the host country [2–4]. Based on the predominance of one or the other, Berry proposes four possible strategies: integration, referring to the individual’s desire to maintain relations with people of the same culture and preserve their customs and habits, while aspiring to maintain relations with members of the dominant culture, while also incorporating the customs and habits of the host country; 2) assimilation, which refers to the rejection of one’s own culture and the desire to relate to the dominant group as a whole; 3) separation, understood as the desire to maintain all the characteristics of one’s own culture while rejecting the dominant culture and relations with its members; and 4) marginalization, in which individuals who feel ambivalent and somehow alienated by both cultures [4].

Several systematic reviews in the last 5 years have evidenced the role of acculturation strategies on both physical and mental health and well-being [5–11], as well as their association with various biomarkers of stress, either inflammatory or endocrine, such as cortisol [12].

Cortisol, commonly known as the stress hormone, is a steroid hormone that serves several functions such as mediating the stress response, regulating metabolism, inflammatory response and immune function [13], so that dysregulations in this hormone are associated with poorer health [14]. Chronic stress is one of the factors most associated with increased basal cortisol concentrations, given its contribution to the dysregulation of the hypothalamic-pituitary-adrenal axis, in addition to affecting the diurnal rhythm of cortisol release [12].

Studies on cortisol in migrant population have been addressed from its relationship with discrimination/ethnicity [15–18], stress derived from the use of a second language [19], trauma experiences during forced migration [20], low social support and high levels of acute stress in refugees [21], family stress [22], coping styles to stress [23], in people fleeing seeking asylum [24], relationship with ethnic/racial identity [25] or from a mediating role in the relationship between acculturation stress and health status [26].

Research linking acculturation or acculturation strategies is still incipient, with mixed results and mainly in the Latino population in the United States. In a study with pregnant Mexican women, it was reported that high levels of acculturation predicted high cortisol levels [27]; in Mexican adolescents it was found that those with a bicultural orientation showed a pattern of high cortisol responsiveness, while those with greater assimilation had an attenuated cortisol response [28]. In migrant college students, mainly Latinos, in the United States, it was similarly reported that bicultural integration would be associated with lower cortisol levels [29].

Given that in the literature reviewed no research has been found on south-south migrants, that is, migrants from a South American country living in a South American country, the present investigation focuses on the Colombian population living in Chile.

This research is located in the commune of Antofagasta, in northern Chile, the region with the largest number of migrants after the Metropolitan Region, with 7% of the total number of migrants in the country. As of 2020, the commune had an estimated migrant population over 18 years of age of close to 51,000 people, of which 40% are estimated to be of Colombian origin [30]. Studies on the Colombian population in Chile and in the city have reported high levels of discrimination, both because of their racial origin (large percentage of Afro-descendants) and their nationality of origin, associated with stereotypes of drug trafficking, violence or sex trade, with negative effects on both their health and well-being [31–39].

Our research group has reported the effect of discrimination on the health of this group, as well as the effect that acculturation strategies may have on various health and well-being outcomes [40–42]. Given that contextual factors are different in migration to an Anglo-Saxon country than to a Latin one (language and culture are close), the aim of this research is to explore the relationship between acculturation strategies and basal cortisol levels in south-south migrants, hypothesizing that the prevalence of a biculturalism strategy could be linked to lower blood cortisol levels.

## Method

This research is part of a larger study that evaluated the effect of discrimination and other variables on the health of migrants. The design used was non-experimental, cross-sectional. Participants completed written questionnaires while waiting their turn for laboratory tests. The laboratory for the analysis of biological samples was certified. The data collection period was between march and december 2021.

### Participants

Inclusion criteria were considered to be migrant, Colombian, over 18 years of age, and to comply with the requirement of arriving at the examination at 08:00 in the morning and then resting for 30 min prior to the blood test, a period of time in which the objective of the research was explained to them and they signed an informed consent form. An additional inclusion criterion was considered to be meeting the requirement of fasting for at least 8 h prior to blood extraction. Women were considered to be pregnant as an exclusion criterion.

Given the impossibility of estimating the size of the sample universe, a non-probabilistic purposive convenience sampling was carried out, using the previously defined inclusion and exclusion criteria for the selection of participants. The recruitment technique was active, i.e., those who met the criteria were directly invited to participate in the study. There was the possibility that participants could suggest other participants who met the same criteria, similar to the respondent driven sampling methodology.

Blood samples were collected and 314 persons completed self-report questionnaires. The samples were collected in the clinical area of the University and in mobile operations carried out in settlements with a high concentration of migrants, such as camps. Participants were compensated US$15.00 for participating in the research.

For the purposes of the analyses, three participants who were taking contraceptives at the time of evaluation were eliminated. Cigarette consumption was recorded, and this variable was then used as a control in the analyses. None of the participants were taking steroid-based medication.

### Measures

#### Cortisol

Cortisol analysis was performed through the enzyme-linked immunosorbent assay technique known as ELISA, which is based on the competitive interaction of cortisol from the sample and an enzyme-hormone conjugate by a limited amount of monoclonal anti-cortisol antibodies [43]. Combiwash equipment (washer, shaker and incubator) was used and its value was determined on the Biotek-800 spectrophotometer, the latter, integrated with GEN5 version 2.09 software, where the results are appreciated. With the addition of the substrate solution a blue color develops, which turns to yellow after stopping the reaction. The intensity of the color is inversely proportional to the cortisol concentration of the sample.

### Acculturation strategies

A Spanish adaptation [44] of the acculturation strategies scale [45] was used. This instrument consists of 21 items with a Likert-type response format from 1 to 5, grouped into 4 dimensions: current practices in the country of origin (8 items); current practices in the host country (3 items); interest in maintaining customs (4 items); and interest in adopting customs (6 items). These 4 dimensions are further combined into 2 dimensions: attitude towards the country of origin and attitude towards the host country. From the combination of valences of both attitudes, 4 acculturation strategies were created: biculturalism-integration, favoring the country of origin and the attitude of the host country; assimilation, showing a positive attitude towards the host country and an unfavorable one towards the country of origin; separation, revealing a positive attitude towards the country of origin and an unfavorable one towards the host country; and marginalization, showing an unfavorable attitude towards both the country of origin and the host country. This scale has been used in other Latin American studies on migrant populations [40–[41, 46]–47], reporting a good fit of the data to the proposed factor structure [42].

### Procedures

The participants were invited to take part in the research at different points in the city, for which they had to come on the day of the blood test, where they also completed the self-report questionnaire.

The extraction process was similar for all participants, the samples being taken between 8:30 and 09:15 am at the latest. The blood samples were transported in an exclusive container for the transport of biological materials, according to the “Technical Regulations for the Transport of Infectious Substances” [48], which must maintain a stable temperature (for plasma cortisol, room temperature) [49] and sent within a maximum of 2 h from sample collection to the Clinical Chemistry laboratory of the Department of Medical Technology of the Universidad de Antofagasta [50]. In order to obtain the serum to be analyzed, the samples were centrifuged at 3,500 rpm for 10 min. A serum sample obtained from the collection of 3.5 ml of peripheral blood was used, using the venous puncture technique in tubes with gel separator and Improvacuter brand coagulation activators, under standardized biosafety regulations version 2018. Samples that presented alterations that prevented their correct biochemical analysis, such as hyperlipemic and/or hemolytic samples, were discarded [51, 52].

To calibrate the ELISA analysis for the quantitative determination of cortisol in human serum or plasma, a calibration curve obtained with the help of calibrators of known cortisol concentrations [CAL]: 0 (A), 20(B), 50 (C), 100 (D), 200 (E), 400 (F) and 800 (G) ng/ml, which are available in the Human Cortisol ELISA kit [REF 55,050]. They are considered valid if the mean absorbance (OD) of [CAL] is greater than or equal to 1.0, in this case the specialist supplier of Farmalatina was consulted and authorized a mean equal to or greater than 0.5. A calibration curve is performed from the absorbances obtained from the calibrators of known concentrations of cortisol with the help of the Biotek program GEN5 version 2.09.

## Analysis

First, the sociodemographic characteristics of the participants were analyzed (see Table 1). Second, descriptive statistics and correlations of the variables of interest were calculated (see Table 2). Third, the data were explored to verify the possible existence of outlier scores on cortisol measures [53]. Fourth, linear regression was performed to estimate the relationship between acculturation orientations and blood cortisol levels controlling for the effects of age, gender, smoking or not, income, and educational level. Fifth, according to Berry’s classification of acculturation strategies [45], participants were grouped into four categories (Integrated, Separated, Assimilated, and Marginalized). For this purpose, the mean of the acculturation orientations was calculated and assigned as a cut-off point to create the categories (see Table 3). Once the four groups were formed, an ANCOVA analysis was performed to compare cortisol levels between the different categories of acculturation strategies controlling for the effects of the effects of age, gender, smoking or not, income and educational level. All statistical analyses were performed using the IBM SPSS version 24 statistical package.

**Table 1:** Sociodemographic characteristics of participants

Sex	
Female	192 (61.6)
Male	122 (38.4)
Self perceived Phenotype *	
White/Caucasic	129 (40.6)
Native	10 (3.1)
Mestizo	69 (21.7)
Afro-descendant	38 (11.9)
Mulatto	26 (8.2)
Others	4 (1.3)
Years of arrival in Chile	
>10 years	27 (8.5)
6–10 years	153 (48.1)
1–5 years	107 (33.6)
Does not respond	31 (9.7)
Education	
No studies	44 (13.8)
Primary education	63 (19.8)
Secundary education	105 (33.0)
Incomplete technical studies	13 (4.1)
Technicians complete	33 (10.4)
Incomplete university studies	12 (3.8)
Complete university studies	6 (1.9)
Does not respond	42 (13.2)
Employment	
Employee	203 (63.8)
Retired	2 (0.6)
Unemployed	33 (10.4)
Housewife	29 (9.1)
Student	17 (5.3) 34 (10.7)
Does not respond	
Monthly income	
<125 US$	29 (9.1)
126–375 US$	111 (33.9)
376–750 US$	109 (34.3)
751-1,250 US$	13 (4.1)
1,251-1,875 US$	2 (0.6)
>1,875 US$	1 (0.3)
Does not respond	53 (16.7)

* *Variables with lost data.*

**Table 2:** Descriptive statistics and correlations between cortisol ng/ml and acculturation orientations

Variables	*n*	Min	Max	Range	Mean	SD	AO	AH
Cortisol ng/ml	313	55	800	745	206.34	125.71	− 0.16*	0.09
Attitudes toward the country of origin (AO)	296	1	5	4	3.57	0.90		− 0.18*
Attitudes toward the host country (AH)	294	1	5	4	2.86	1.02		

**Table 3:** Multiple regression analysis where Attitudes towards country of origin and Attitudes towards host country predict blood cortisol levels

		Non-standardized coefficients		Standardized coefficients	*t* (Sig.)
Block		B	Standard error	Beta	
1	Constant	257.630	33.316		7.733 (0.000)
	Age	− 0.092	0.456	− 0.014	− 0.201 (0.841)
	Sex	-19.029	11.234	− 0.120	-1.694 (0.092)
	Smoker	-28.048	15.961	− 0.125	-1.757 (0.080)
	Income	-13.464	6.875	− 0.142	-1.958 (0.052)
	Education	-1.982	3.759	− 0.038	− 0.527 (0.599)
2	Constant	313.364	43.679		7.174 (0.000)
	Age	− 0.009	0.461	− 0.001	− 0.020 (0.984)
	Sex	-20.885	11.196	− 0.131	-1.865 (0.064)
	Smoker	-26.946	15.906	− 0.120	-1.694 (0.092)
	Income	-14.660	6.894	− 0.154	-2.126 (0.035)
	Education	− 0.819	3.793	− 0.016	− 0.216 (0.829)
	AO	-14.409	6.659	− 0.148	-2.164 (0.032)
	AH	-1.438	5.397	− 0.018	− 0.266 (0.790)

*Note*: AO = Attitudes towards country of origin; AH = Attitudes towards host country.

## Results

### Participants

The sample consisted of 314 participants (Table 1). Of these, 192 (61.6%) were women and 122 (38.4%) were men. The mean age of the participants was 35 years (men: 34.11, SD = 12.56; women: 35.56, SD = 11.33). The majority of participants defined themselves as white (n = 129, 40.6%) or mixed race (n = 69, 21.7%). Of the participants, 81.7% (n = 260) arrived in the country less than 10 years ago. In terms of educational level, the majority of participants reported having completed high school (n = 105, 33%). In the case of employment, 63.8% (n = 203) of the participants are currently working. 68.2% (n = 120) of the respondents reported earning between US$126 and US$750 per month. The waking hours of each participant were not controlled. In addition, 11.4% (n = 33) of the participants reported current smoking.

### Cortisol and acculturation strategies

Table 2 shows the descriptive statistics and correlations between cortisol ng/ml and acculturation orientations. As has been shown, an inverse relationship is observed between the preference for preserving the culture of the country of origin and cortisol levels, that is, the greater the tendency to preserve one’s own culture, the lower the levels of cortisol ng/ml in the blood. No statistically significant correlation was detected between the preference to adopt part of the host country culture with blood cortisol nl/ml.

Before continuing with the following analyses, the data were explored through box plots in order to identify outliers in the cortisol measurement scores. Outliers were considered to be scores more than 1.5 interquartile ranges away from the 75th percentile [54]. Outliers were iteratively extracted until there were no extreme scores. Using this method, 22 outliers were discarded in subsequent analyses.

Once the outliers were discarded, multiple linear regression was calculated to predict blood cortisol levels from acculturation orientations (AO and AH). To proceed with the multiple regression analysis, we first tested for noncollinearity of the predictor variables by tolerance indices and variance inflation factors (VIFs). Tolerance values less than 0.10 and VIF values greater than 10 would indicate problems of collinearity between the predictors of the model [55]. However, in our case, AO presented a tolerance value equal to 0.976 and a VIF value equal to 1.025, while AH, presented a tolerance value equal to 0.957 and a VIF value equal to 1.045. Therefore, no values indicative of collinearity were detected and we can continue with the regression analysis.

In the multiple regression, the first block included age, sex, smoking or not, income and education. In the second block, Attitudes towards country of origin and Attitudes towards host country were added. The model grouping the control variables did not present a significant regression equation (F (5, 208) = 1.917, p = .093, with an R² of 0.044 (R = .210). While the model containing the acculturation orientations did present a significant regression equation (F (7, 206) = 2.057, p = .049, with an R² of 0.065 (R = .256). As can be seen in Table 3, Attitudes towards country of origin was a statistically significant predictor (p = .032) of blood cortisol. However, in the case of Attitudes towards host country, these were not a statistically significant predictor (p = .790) of the dependent variable.

After performing the regression analysis, we proceeded to create the four categories of Berry’s acculturation strategies. Table 4 presents the sample means of cortisol ng/ml for each of the acculturation strategies. In addition, the number and percentage of migrants included in each of the categories can be observed.

**Table 4:** Percentage of participants in each acculturation strategy and their sample averages for cortisol ng/ml

Acculturation Strategy	*n* (%)	*Me (SD)*
Integrated	63 (21.3)	153.14 (61.92)
Separated	110 (37.2)	187.47 (79.55)
Assimilated	49 (16.6)	208.83 (80.58)
Marginalized	74 (25.0)	170.92 (81.68)
Total	296 (100)	180.82 (78.39)

Before performing the ANCOVA, cortisol was tested for normality and homoscedasticity for each of the acculturation strategies. According to the Kolmogorov-Smirnov test, the scores of the integrated (*p* = .200), assimilated (*p* = .200) and marginalized (*p* = .076) were normally distributed, but not in the case of the assimilated (*p* = .003). As for homoscedasticity, according to Levene’s test, the error variance of the dependent variable is equal between groups (*F* = 1.316, *p* = .270).

**Fig. 1:**
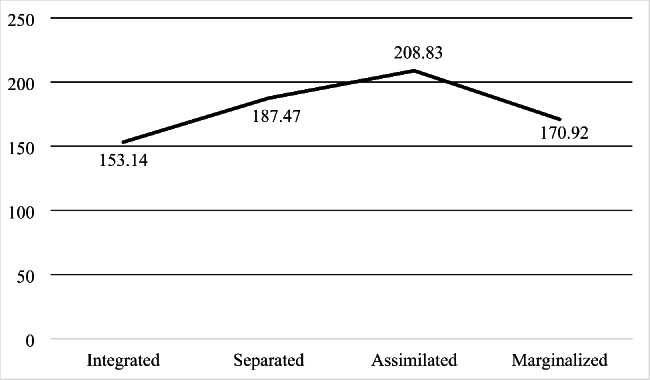
Median cortisol in each of the acculturation strategies

Once the normality and homoscedasticity of the variables had been proven, ANCOVA was performed. The results showed the existence of statistically significant differences in cortisol between the different types of acculturation strategies, after controlling for age, sex, smoking or not, income and educational level (*F* (3, 208) = [4.398], *p* = 005). According to multiple comparisons (see Fig. 1), the mean cortisol value was significantly different between integrated and assimilated subjects (*p* = .003, 95% CI = [-102.61, -13.89]), with the mean cortisol of the integrated being significantly lower than that of the subjects typed as marginalized. No statistically significant differences were observed between integrated and separated subjects (*p* = .092, 95% CI = [-71.26, 3.06]), between integrated and marginalized (*p* = 1.000, 95% CI = [-66.05, 23.33]), between separated and assimilated (*p* = .604, 95% CI = [-63.17, 14.87]), and between separated and marginalized (*p* = 1.000, 95% CI = [-25.92, 51.39]).

## Discussion

The aim of this research was to analyze the relationship between acculturation strategies and blood cortisol levels in the south-south migrant population. Our initial hypothesis was that those immigrants who presented a bicultural (integrated) orientation, that is, who were able to coexist with both their culture of origin and that of the host country, would have lower cortisol levels. This result was expected according to studies carried out in the Latino population in an Anglo-Saxon country [7]. The results obtained provide evidence that those who opt for a bicultural strategy have lower cortisol levels than those who opt for an assimilation option, even controlling for variables that could affect cortisol levels, such as age, sex, smoking, income and educational level. Similarly, cortisol levels are lower in those who opt for an integration strategy than for separation and marginalization strategies, these differences are not statistically significant.

The group of individuals using the integration strategy showed the lowest cortisol levels, with significantly lower values than the group that opted to assimilate into the host country culture. Although most of the evidence on the use of acculturation strategies suggests that biculturalism (integration) and marginalization are associated reports that question these findings. For example, no differences in mental health have been reported in immigrant youth in different continents using the integration and marginalization strategy [56]. While another study on young children of Turkish immigrants living in the United Kingdom reported no relationship between marginalization and integration strategies and mental health [57].

Even, in certain situations marginalization may be associated with better health. In one of the first studies we conducted, we related mental health and acculturation strategies in a Colombian and Peruvian population living in Chile [41]. Consistent with the literature, we found that those who opted for the marginalization strategy (in the Colombian case) and assimilation (in the Peruvian case) presented greater anxious and depressive symptomatology compared with the other strategies. However, when evaluating difficulties in interpersonal relationships, Colombian and Peruvian migrants who opted for marginalization and separation, respectively, presented fewer difficulties than those who used other strategies. Likewise, the Colombian and Peruvian immigrant community that used marginalization perceived fewer difficulties in social role adjustment than those who opted for assimilation or integration strategies. Thus, Urzúa’s study [41] shows that the marginalization strategy could be associated with positive results in certain areas. In this line, a possible explanation for what was found in the present study is that marginalization would not require cognitive or behavioral efforts to try to coexist with the culture of origin and of the host country, so that stress is lower, hence, the cortisol response is also lower.

Similarly, it was observed that immigrants who show a preference for preserving the culture of their country of origin have lower levels of cortisol ng/ml in blood. This finding may be interesting, given that as people prefer less to preserve their own culture, their cortisol levels increase. This could be associated with the wear and tear that the person must perform in biological terms to try to preserve his or her identity in the face of a majority culture, especially in contexts in which the immigrant population perceives discrimination towards their culture of origin by the host country. In this sense, significant levels of discrimination by the Chilean population toward immigrants in general [58] and toward the Colombian and Peruvian communities [59–62] have been reported. This discrimination is partly due to the fact that these communities are perceived as a threat to the culture and identity of the host country. Despite these effects on stress, the permanence of this effort to reinforce ethnic identity can be explained by the protective factor it has on mental health [63–66].

The findings of this study open several avenues of research on the relationship between acculturation strategies and cortisol. It appears that the biculturalism-cortisol association does not necessarily apply to all immigrant groups or migration contexts, but that marginalization may work well in some. Likewise, although ethnic identification tends to protect the individual’s mental health or lower his or her stress (and thus cortisol) levels, this process could act as a protective factor in certain phases of the migratory process and in a specific context characterized by high discrimination. Future studies could address this suggestion by considering the degree of discrimination of immigrants in the host society and by assessing different domains associated with mental health. The latter will make it possible to examine the differential results according to the domain assessed noted by Urzúa [41].

Other future lines of research arise from the limitations of this study. One limitation is that the relationship between acculturation strategies and cortisol was examined considering one migrant group, so the results cannot be directly extrapolated to others. Thus, it is necessary to analyze the behavior of this relationship in other migrant populations and host societies, for two reasons. First, because the relationship between acculturation strategies and cortisol would depend on the levels of integration and acceptance by the majority society, since this could enhance or hinder certain acculturation strategies. Second, because the degree of complementarity or integration that may occur between the two cultures would depend on the values of the immigrant community, such as its individualistic or collectivist orientation, as has been previously reported [5, 67].

A second limitation is that the relationship is shown at a specific point in time, but we cannot infer how it evolves over time. This requires further elaboration through longitudinal studies to assess the relationship as the person becomes more integrated and has greater contact with the host population. Such studies would show the relevance of the acculturation strategy at different points in the integration of the immigrant community.

Despite these limitations, this study enriches the literature on the relationship between acculturation strategies and cortisol by providing evidence that, resembling from most previous studies, opens new questions that encourage future more detailed analyses of both variables.

## Conclusion

The patterns of the relationship between biculturalism and cortisol found in Latino migrants in Anglo-Saxon countries are repeated in Latino migrants in a Latino country. The influence of other variables on this relationship, such as the effect of discrimination or the presence of specific collective coping strategies, needs to be explored, as it seems that the best adaptive strategy, and therefore the cortisol response, will vary according to the sociocultural context of the host country.

In addition, this study provides evidence of the relationship between psychological variables that can affect biological markers in the migrant population, generating theoretical support for the development of interventions and policies that can contribute to protect the health of the migrant population, in this case, favoring the maintenance of the culture of origin as a protective factor, in a transition towards a strategy of biculturalism.

## Data Availability

The data presented in this study are available on request from the corresponding author. The data are not publicly available because the project has state funding and will only be released once the project is finished.
